# De novo deletion of chromosome 11q12.3 in monozygotic twins affected by Poland Syndrome

**DOI:** 10.1186/1471-2350-15-63

**Published:** 2014-05-30

**Authors:** Carlotta Maria Vaccari, Maria Victoria Romanini, Ilaria Musante, Elisa Tassano, Stefania Gimelli, Maria Teresa Divizia, Michele Torre, Carmen Gloria Morovic, Margherita Lerone, Roberto Ravazzolo, Aldamaria Puliti

**Affiliations:** 1 Department of Neurosciences, Rehabilitation, Ophthalmology, Genetics, Maternal and Child Health (DiNOGMI), University of Genoa, Genoa, Italy; 2 Plastic and Reconstructive Surgery Unit, IST - San Martino Hospital and University of Genoa, Genoa, Italy; 3 Medical Genetics Unit, Istituto Giannina Gaslini, via G. Gaslini 5, 16148, Genoa, Italy; 4 Service of Genetic Medicine, University Hospitals of Geneva, Geneva, Switzerland; 5 Pediatric Surgery Unit, Istituto Giannina Gaslini, Genoa, Italy; 6 Pediatric Plastic Surgery Unit, Dr. Luis Calvo Mackenna Hospital, Santiago, Chile; 7 CEBR, University of Genoa, Genoa, Italy

**Keywords:** Chromosome 11q deletion, Congenital abnormalities, Monozygotic twins, Poland syndrome, CNV, *HRASLS5*, *HRASLS2*, *RARRES3*, *PLA2G16*

## Abstract

**Background:**

Poland Syndrome (PS) is a rare disorder characterized by hypoplasia/aplasia of the pectoralis major muscle, variably associated with thoracic and upper limb anomalies. Familial recurrence has been reported indicating that PS could have a genetic basis, though the genetic mechanisms underlying PS development are still unknown.

**Case presentation:**

Here we describe a couple of monozygotic (MZ) twin girls, both presenting with Poland Syndrome. They carry a *de novo* heterozygous 126 Kbp deletion at chromosome 11q12.3 involving 5 genes, four of which, namely *HRASLS5*, *RARRES3*, *HRASLS2*, and *PLA2G16*, encode proteins that regulate cellular growth, differentiation, and apoptosis, mainly through Ras-mediated signaling pathways.

**Conclusions:**

Phenotype concordance between the monozygotic twin probands provides evidence supporting the genetic control of PS. As genes controlling cell growth and differentiation may be related to morphological defects originating during development, we postulate that the observed chromosome deletion could be causative of the phenotype observed in the twin girls and the deleted genes could play a role in PS development.

## Background

Poland Syndrome (PS) (OMIM 173800) is characterized by hypoplasia/aplasia of the pectoralis major muscle, variably associated with thoracic and upper limb anomalies. Thoracic anomalies in PS include breast and rib hypoplasia/aplasia, sternal anomalies, mammary gland anomalies, and limited subcutaneous fat. Upper limb anomalies can be absent or as severe as phocomelia-like anomalies. The classic hand deformity in PS includes syndactyly and variable degrees of brachydactyly with hypoplasia or aplasia of the middle phalanges (reviewed in [1,2]). The incidence of PS was reported to be 1/30,000-32,000 births with higher prevalence in males and a male to female ratio estimated between 2:1 and 3:1 [3-5]. The pathogenic mechanisms underlying PS are still unknown. Indeed, the genetic origin of the disease is still a matter of debate. One hypothesis states that PS defects could result from a vascular insult during early embryological stages, which implies that environmental factors could contribute to PS phenotype [6,7]. However, though most cases of PS are sporadic, familial recurrence with higher prevalence in males has been observed, which suggests a genetic basis of this congenital anomaly [3]. Different inheritance patterns, including autosomal recessive, autosomal dominant, and dominant with incomplete penetrance, have been reported and, therefore, genetic heterogeneity might be expected (reviewed in [3]). Impaired development of structures involved in PS may be hypothesized, suggesting a pathogenic mechanism involving genes regulating cell proliferation and/or differentiation during development. In agreement with this view, it has been recently proposed that all forelimb elements, which include skeletal structures, proximal and distal muscles, and sternum, develop according to a unique program [8].

Here we describe a couple of MZ twin girls with PS, in whom a deletion at chromosome 11q12.3 was identified by array-comparative genomic hybridization (array-CGH). The deletion involved 5 genes, namely: *HRASLS5, LGALS12, RARRES3, HRASLS2,* and *PLA2G16*, among which four are members of the HREV107 type II tumor suppressor gene family encoding proteins that regulate cellular growth, differentiation, and apoptosis.

## Case presentation

The twin patients are daughters of healthy non-consanguineous parents from Santiago, Chile. No exposure to drugs, alcohol, and infections was reported during pregnancy; the mother was hospitalized three times for threatened abortion. The patients were born full term after spontaneous delivery. Birth weight was respectively 2.500 kg (first twin) (5th centile) and 2.000 kg (second twin) (<3rd centile), length was 45 cm (first twin) (3rd centile) and 40 cm (second twin) (<3rd centile). Both twins were kept in the neonatal unit because of low birth weight. The first twin had a normal neonatal period, while the second showed severe asphyxia at birth. Psychomotor development and growth were normal in both twins. The parents reported that the toracic anomaly was well observable in both girls since the age of 10 years. At the age of four years, the first twin underwent surgery because of a thyroglossal duct cyst that, to the best of our knowledge, has never been previously reported in PS patients. At twelve years, she presented mild scoliosis without indication for surgical intervention. The second twin had severe congenital scoliosis, due to multiple vertebral malformations (T4 hemivertebra and T5-T6 butterfly vertebrae), treated surgically when she was thirteen years old. Vertebral anomalies, although not common in PS, have already been reported in some cases [9] and were also present in another patient of our “independent PS control group” (see below). At 13 years, the twin girls were admitted to the Luis Calvo Mackenna Hospital (Santiago, Chile) because of breast asymmetry. Pectoral muscle, breast, and mild axillary fold hypoplasia on the right side was clinically observable in both patients, more evident in the first twin. Ultrasound examination revealed, in both patients, an important breast and pectoralis major muscle hypoplasia on the right side, thus confirming the diagnosis of PS. At that time, both twin girls were referred for genetic counseling to the Genetic Unit at Luis Calvo Mackenna Hospital. The clinical geneticist did not observe any additional associated dysmorphic features, normally present in other disorders similar to PS, as anomalies of hair, teeth, nails, skin, vision, hearing, or neurologic aspects. Therefore, other associated malformations and syndromes were excluded in both patients.

At the age of 19 years, the first twin was 1.59 m in height (25^th^ centile), 52.3 Kg in weight (25^th^-50^th^ centile); the second twin was 1.53 m in height (5^th^ centile), 46.7 Kg in weight (5^th^-10^th^ centile).

**Figure 1:**
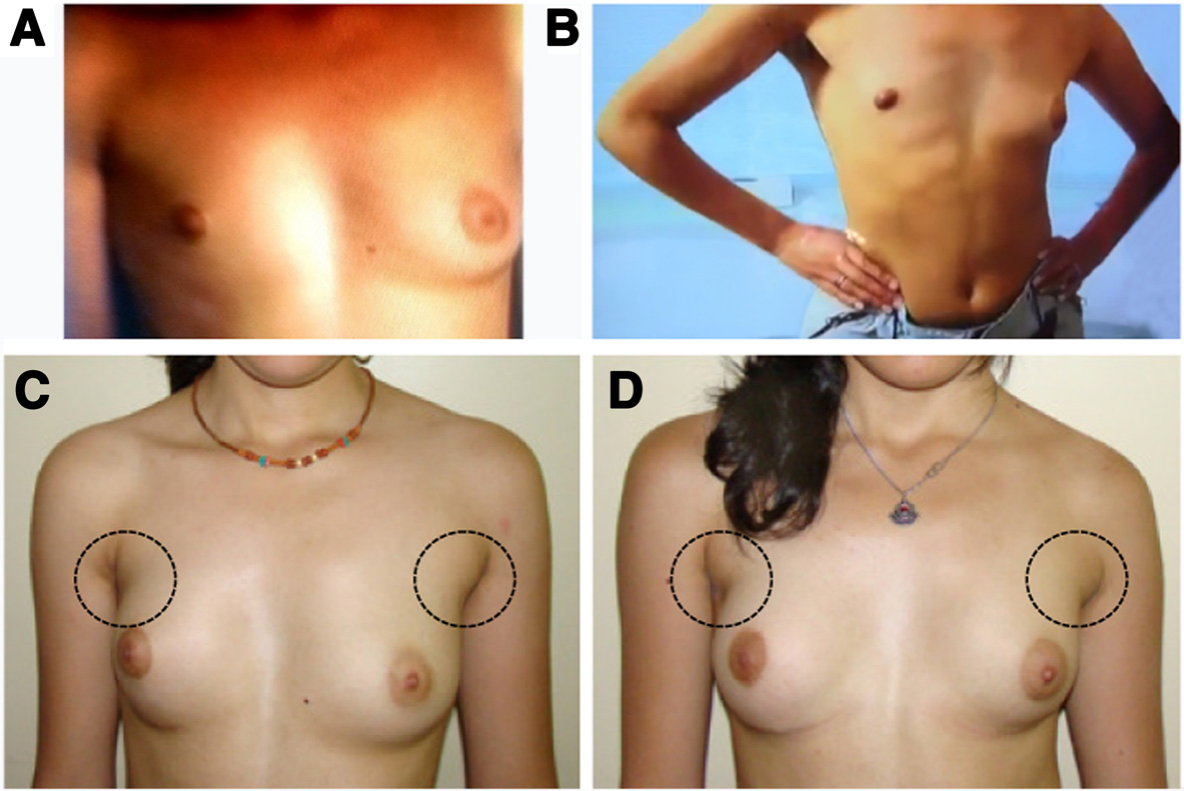
**Chest images from the twin girls. A and C)** First twin. **B and D)** Second twin. **A and B)** Breast asymmetry, depression of the anterior chest wall indicating pectoral muscle hypoplasia, cranially located nipple, and hypoplastic areola are visible on the right side of both twin girls before surgery. **C and D)** Slight asymmetry and pectoral muscle hypoplasia are still perceivable, more evident in the first twin (left), two years after surgery.

**Figure 2:**
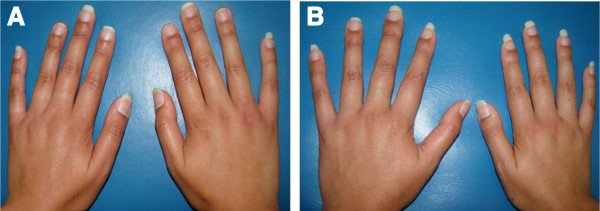
**Hands of the twin girls at the age of 19 years.** Hand anomalies are not evident in any of the two patients. **A)** First twin’s hands. **B)** Picture of the second twin’s hands showing slight hypoplasia of the right one.

The thoracic anomaly was surgically treated in both girls at 17 years of age according to already established protocols [10] (Figure 1). The preoperative evaluation included contralateral pectoral and breast measurements for the choice of the most appropriate breast or pectoral implant, i.e., best reproducing the normal side of the patient in size, volume, and shape. Indeed, both twin girls underwent echocardiographic and abdominal ultrasonographic examinations which excluded the presence of any congenital heart and abdominal abnormalities. X-ray examination excluded the presence of skeletal anomalies, other than the vertebral anomalies of the second twin, and/or skeletal dysplasia reminiscent of other disorders. In the second twin, slight hypoplasia of the right hand was observable (Figure 2). Hand length, measured from the tip of the distal phalanx of the middle finger to the distal crease of the wrist with the hand in the neutral flexion/extension pose, was 17.7 cm for the left hand and 16.8 cm for the right hand. The first twin had symmetric hands (18.3 cm hand length). Neither patients exhibited any dysmorphic hand anomalies (Figure 2). In parents and siblings (one sister and one brother, both younger than the twin girls), PS was excluded on the basis of clinical evaluation.

A cohort of additional 30 unrelated sporadic PS patients (19 males and 11 females), defined as “independent PS control group”, was included in this study for further molecular investigations. Seventeen patients presented right-sided PS (9 males and 8 females). Twelve cases had isolated pectoral muscle hypoplasia/aplasia. Eighteen patients presented with various associated skeletal defects, namely: pectus excavatum (n = 1); rib anomalies (n = 3); vertebral defects (n = 1); craniosynostosis (n = 1); upper limb anomalies (n = 14).

## Methods

### Genotyping

To investigate the twins’ zygosity, microsatellite analysis was performed on genomic DNA extracted from each twin’s blood, using 9 short tandem repeat (STR) loci (*D3S1358*, *vWA*, *FGA*, *TH01*, *TPOX*, *CSF1PO*, *D5S818*, *D13S317*, and *D7S820*) and the Amelogenin locus (AmpFLSTR® Profiler® PCR Amplification Kit, Life Technologies, Foster City, CA, USA).

### Array comparative genomic hybridization (Array-CGH)

DNA from blood lymphocytes was obtained from twins, healthy parents, and siblings. The array-CGH analysis was performed using Human Genome CGH Microarray Kit G3 180 (Agilent Technologies, Palo Alto, USA) with ~13 Kbp overall median probe spacing. Labelling and hybridization were performed following the protocols provided by the manufacturers. A graphical overview was obtained using the Agilent Genomic Workbench Lite Edition Software 6.5.0.18.

Aberration segments were reviewed using GRCh37 (February 2009) hg19 of UCSC Genome Browser [11]. DGV (Database of Genomic Variants) [12], DECIPHER Database [13], and ISCA Consortium database [14] were also used.

### Validation of copy number variations (CNVs)

Array-CGH results were validated by loss of heterozygosity (LOH) analysis and real-time PCR (qPCR). For LOH analysis, DNA from probands and their parents was amplified and sequenced using primers specific for four SNP markers in the deleted region, namely rs4088473, rs637122, rs7104097, and rs4088472. For qPCR, DNA from both twin patients and their parents was analyzed, as well as DNA from one healthy adult used as control. Primers were designed to amplify a region encompassed by the deletion (specific for the *HRASLS5* gene, chr11:63,241,331-63,241,440), a 5′ flanking region (specific for *SLC22A*, chr11:63,144,692-63,144,838), and a 3′ flanking region (specific for *PLA2G16*, chr11:63,375,848-63,375,956). A region on chromosome 12 encompassing the *GAPDH* gene (NM_002046.3) was used as an internal control to determine copy number and normalize primer efficiency (primer sequences are available on request). qPCR was performed using the iCycler (Biorad, Hercules, CA) with Sybr Green and the comparative DDCt method [15].

### Sequence variation analyses

All 5 genes, either completely or partially encompassed by the deletion, were screened in a series of sporadic cases (independent PS control group), namely: *HRASLS5, LGALS12, RARRES3, HRASLS2, PLA2G16*. The entire coding sequence of these genes and their flanking intronic regions were amplified by PCR on genomic DNA (primer sequences are available on request), purified, and then sequenced on both strands using BigDye dideoxy-terminator chemistry on an ABI 3100 DNA sequencer (Applied Biosystems, Foster City, CA). Amino acid change effects were predicted with the PolyPhen-2 program [16], and effects on splicing with the BDGP software (The Berkeley Drosophila Genome Project) [17,18]. To characterize regions enriched in functional elements, we performed comparisons of orthologous genomic DNA sequences using the GERP (Genomic Evolutionary Rate Profiling) program [19]. Variant results were reviewed using dbSNP [20] and NHLBI ESP Exome Variant Server [21].

## Molecular study results

**Figure 3:**
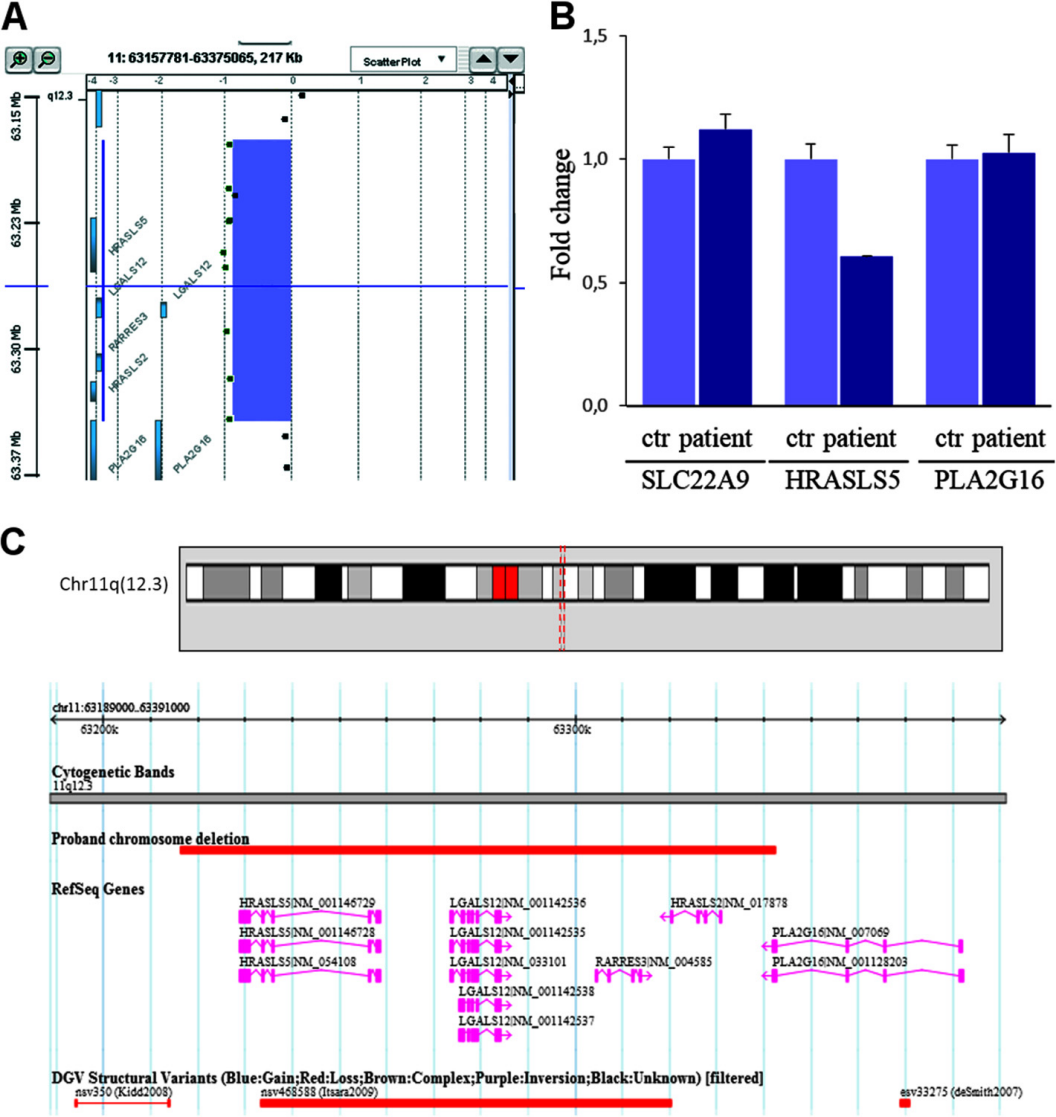
**Identification of a de novo chromosome 11q12.3 deletion in MZ twins with Poland Syndrome. A)** Array-CGH profile of chromosome 11 deleted segment. **B)** qPCR results for a region encompassed by the deletion (specific for the *HRASLS5* gene), a 5′ flanking region (specific for *SLC22A* gene), and a 3′ flanking region (specific for *PLA2G16* gene) obtained in the proband and one control individual. The copy number changes were calculated using the comparative DDCt method. Fold change of about 1 is expected for a diploid sample, and of about 0.5 for a haploid sample. **C)** Screenshot of chromosome 11 corresponding to the genomic position (chr11:63,216,306-63,342,369, NCBI build 37). Patient chromosome deletion (upper red bar), RefSeq genes, and Copy number variations (based on DGV, filtered to show only deletions) in the studied region.

The DNA microsatellite analysis demonstrated that the twin girls are identical. The CGH microarray analysis showed a heterozygous 126 Kbp interstitial deletion of chromosome 11q12.3, arr [hg19] 11q12.3(63,216,306-63,342,369)x1, in both patients (Figure 3A). The array-CGH analysis showed that the deletion was not present in the probands’ parents and healthy siblings, which indicated a *de novo* origin. The patients’ CNV was validated by qPCR (Figure 3B) and LOH analysis, which also showed that the deletion occurred on the paternal chromosome. The deletion spans the entire sequence of the following four genes: *HRASLS5, LGALS12, RARRES3, HRASLS2*, and, distally, *PLA2G16* in its terminal part (Figure 3). The coding regions of the 5 genes were sequenced to exclude the presence of deleterious and/or rare variants in the non-deleted homologous chromosome of both twins. No identical CNV was found in controls from the DGV database and in our 200 internal controls except for a smaller CNV in one control (Figure 3C) and a partially overlapping CNV in another control encompassing *HRASLS5*, *LGASL12*, and *RARRES3* genes. Similar CNVs related to specific disorders or intellectual disability are not included in the ISCA database. A larger deletion (about 2.6 Mbp) overlapping the probands’ one in a patient with intellectual disability and apparently no other associated anomalies is included in the Decipher database. No mutations affecting any of the screened genes have ever been reported associated with any known disorders.

In the independent PS control group, overall 8 variants, distributed among 3 out of the 5 screened genes, were found (Table 1). Three are missense variants; one is a synonymous variant, and four are in exon-flanking intronic sequences; none of these four seemed to alter gene splicing. All of them were previously reported in variant databases except for one missense heterozygous variant affecting *HRASLS5* (chr11:63,258,424, C > T; Arg28Gln, Table 1) in one patient. The genomic position of this variant did not seem to be very conserved (GERP score = −5.14); pathogenicity prediction evaluated by PolyPhen2 suggested a possible damaging effect (0.897 score). This variant was transmitted to the patient by his healthy father.

**Table 1:** Rare gene variants identified in the “independent PS control group”

**Gene**	**Position (hg19)**	**Ref**	**Alt**	**MAF**	**Variants**	**dbSNP id**	**Freq**	**Pathogenicity**	
								**GERP**	**POLYPHEN2 prediction**
HRASLS5	chr11:63,230,659	G	C	0.040	Intron_variant	rs2282479	0.017	1.14	Unknown
HRASLS5	chr11:63,233,710	A	G	0.189	Leu207Leu	rs2275999	0.100	4.16	Unknown
HRASLS5	chr11:63,256,441	C	G	0.207	Ala93Pro	rs940611	0.033	−1.03	Benign (0.000)
HRASLS5	chr11:63,258,424	C	T	unknown	Arg28Gln	unknown	0.017	−5.14	Poss-dam (0.897)
LGALS12	chr11:63,276,480	G	A	0.023	Intron_variant	rs200256001	0.033	−2.69	Unknown
LGALS12	chr11:63,277,334	A	G	0.009	Ile176Val	rs117587231	0.017	4.42	Benign (0.005)
LGALS12	chr11:63,278,621	C	G	0.333	Intron_variant	rs2239679	0.200	−0.07	Unknown
PLA2G16	chr11:63,381,458	C	T	0.072	Intron_variant	rs61929725	0.150	−5.26	Unknown

Variant location is reported using hg19 coordinates. MAF, minor allele frequency according to the NCBI dbSNP137 database; Freq, frequency of the variant allele in the cohort of 30 patients; GERP, Genomic Evolutionary Rate Profiling; the POLYPHEN2 measures of pathogenicity are: Benign, possibly damaging (Poss-dam), and probably damaging based on the false discovery rate.

## Discussion

PS has been hypothesized to have a genetic origin. Here we describe a couple of MZ twin girls both presenting with PS. Phenotype concordance in monozygotic twins affected by a disease is generally accepted as evidence supporting the hypothesis that this disease is under genetic control. In this view, the fact that both our twin girls exhibited pectoral muscle hypoplasia might contribute to support the hypothesis of a genetic control of PS.

Both twins exhibited skeletal anomalies, one of the two showing a more severe phenotype. Discordance in disease manifestation between affected monozygotic twins has already been reported and attributed to different patterns of X chromosome inactivation, epigenetic mechanisms involving differences in methylation patterns, somatic mutations casually occurring during development, or environmental factors [22-24]. The differences in the phenotype of our twin girls could otherwise be related to the effects of vascular interruptions during their embryonic life due to thrombosed microvasculature caused by predisposing genetic factors, as also recently proposed to explain the origin of PS in a sporadic case [25].

Notably, PS was previously reported in only one of two identical twin girls [26], indicating the complexity of genetic mechanisms underlying PS. Our twin girls share a heterozygous 126 Kbp interstitial deletion at chromosome 11q12.3 spanning 5 genes. This specific deletion, not known to be associated with any specific genetic disorders, could be considered as a candidate region having a role in PS development. We investigated the function and expression of the 5 genes with the aim to explore their involvement in patients’ clinical manifestations.

All forelimb elements, including skeletal structures, proximal and distal muscles, and sternum, seem to develop according to a unique program [8]. Thus, all tissues involved in PS could develop under the control of the same group of genes whose mutations may account for the various features characterizing the PS phenotype. According to this hypothesis, we considered as less likely candidate genes those lacking a clear role in the development of tissues involved in PS, as *LGALS12*, which encodes a member of the beta-galactoside-binding lectin family expressed by adipocytes.

The remaining four genes, namely *HRASLS5, RARRES3, HRASLS2,* and *PLA2G16*, are members of the HREV107 type II tumor suppressor gene family known to regulate cellular growth, differentiation, and apoptosis, mainly through the Ras-mediated signaling pathways [27]. In particular, *RARRES3*, a retinoid-inducible growth regulator, was shown to reduce the level of activated Ras [28]. *HRAS* activation was reported to inhibit skeletal myogenesis by favoring proliferation of myoblasts and by blocking their differentiation into muscle cells [29-32]. For these reasons, one may speculate that, in our patients, the heterozygous deletion of *RARRES3* suppressor gene could have induced an enhancement of *HRAS* expression and activity interfering with correct skeletal muscle differentiation.

We searched for mutations in all genes involved in the deletion in a panel of 30 PS patients. Rare variants with no clear direct causative effects were found. Only one unreported variant was found in one patient, a missense variant in the *HRASLS5* gene (Arg28Gln) transmitted to the PS patient by his healthy father. Thus, it is reasonable to exclude a direct causative role in PS of this variant, that could rather act as a predisposing factor. On the one hand, absence of mutations in a relatively small cohort of patients suggests that the analyzed genes are not frequently involved in PS, on the other hand, the question whether the detected rare variants could exert a modifying effect on the PS phenotype is still controversial.

## Conclusions

In conclusion, PS phenotype concordance in our monozygotic twin girls might represent an important evidence supporting the genetic control of PS. The twin patients shared a heterozygous chromosome 11q12.3 deletion including genes (*HRASLS5, RARRES3, HRASLS2,* and *PLA2G16*) involved in cellular growth, differentiation, and apoptosis through Hras signaling pathways. As PS may result from impaired development, genes controlling cell growth and differentiation may represent good candidate genes. In this view, we postulate that the deletion of one copy of these genes could be causative of PS phenotype in the twin girls, although future studies are warranted to clarify which proportion of PS patients could be accounted for by mutations in these genes.

### Consent

This study was approved by the Ethics Committee of the Giannina Gaslini Institute (Protocol number: RR_AP IGG 001; Title: Identification of genetic bases of Poland Syndrome). Written informed consent for the collection of peripheral blood samples and subsequent analyses was obtained from all participating families, with the parents giving consent for themselves and on behalf of their minor children. Permission for publication of the images was obtained from the twin patients. A copy of the written consent is available for review by the Editor-in-Chief of this Journal.

## Abbreviations

CGH: Comparative genomic hybridization; CNV: Copy number variation; LOH: Loss of heterozygosity.

## Competing interests

The authors declare that they have no competing interests.

## Authors’ contributions

AP and RR conceived the study and participated in its design and coordination; CMV, MVR, MTD, MT, CGM, ML identified the patients and carried out the clinical characterizations; ET and SG carried out the array-CGH analyses; CMV and IM performed the qPCR and gene screening; AP wrote the manuscript; all authors read and approved the final manuscript.

## Pre-publication history

The pre-publication history for this paper can be accessed here:

http://www.biomedcentral.com/1471-2350/15/63/prepub
